# Supplementary material for: Resolving the full spectrum of human genome variation using Linked-Reads
Source: Genome Res. 2019 Apr;29(4):635–45. doi: 10.1101/gr.234443.118 (PMC6442396; doi:10.1101/gr.234443.118)
Supplement: Supplemental Material [file supp_gr.234443.118_Supplemental_longranger-2.2.zip › longranger-2.2.tar.bz2!/lr2.2-public/lib/stan/cmdstan/stan/doc/api/html/program__grammar_8hpp_source.html]

Stan: src/stan/gm/grammars/program\_grammar.hpp Source File


|  |  |
| --- | --- |
|  | Stan  2.5.0  probability, sampling & optimization |


- Main Page
- Related Pages
- Namespaces
- Classes
- Files

- File List
- File Members

All Classes Namespaces Files Functions Variables Typedefs Enumerations Enumerator Friends Macros Pages

- src
- stan
- gm
- grammars

program\_grammar.hpp

Go to the documentation of this file.

1 #ifndef STAN\_\_GM\_\_PARSER\_\_PROGRAM\_GRAMMAR\_\_HPP

2 #define STAN\_\_GM\_\_PARSER\_\_PROGRAM\_GRAMMAR\_\_HPP

3

4 #include <string>

5 #include <sstream>

6 #include <vector>

7

8 #include <boost/spirit/include/qi.hpp>

9

10 #include <stan/gm/ast.hpp>

11 #include <stan/gm/grammars/whitespace\_grammar.hpp>

12 #include <stan/gm/grammars/expression\_grammar.hpp>

13 #include <stan/gm/grammars/var\_decls\_grammar.hpp>

14 #include <stan/gm/grammars/statement\_grammar.hpp>

15 #include <stan/gm/grammars/functions\_grammar.hpp>

16

17 namespace stan {

18

19  namespace gm {

20

21  template <typename Iterator>

22  struct program\_grammar

23  : boost::spirit::qi::grammar<Iterator,

24  program(),

25  whitespace\_grammar<Iterator> > {

26

27  program\_grammar(const std::string& model\_name);

28

29  // global info for parses

30  std::string model\_name\_;

31  variable\_map var\_map\_;

32  std::stringstream error\_msgs\_;

33

34  // grammars

35  expression\_grammar<Iterator> expression\_g;

36  var\_decls\_grammar<Iterator> var\_decls\_g;

37  statement\_grammar<Iterator> statement\_g;

38  functions\_grammar<Iterator> functions\_g;

39

40  // rules

41

42  boost::spirit::qi::rule<Iterator,

43  std::vector<var\_decl>(),

44  whitespace\_grammar<Iterator> >

45  data\_var\_decls\_r;

46

47  boost::spirit::qi::rule<Iterator,

48  std::pair<std::vector<var\_decl>,

49  std::vector<statement> >(),

50  whitespace\_grammar<Iterator> >

51  derived\_data\_var\_decls\_r;

52

53  boost::spirit::qi::rule<Iterator,

54  std::pair<std::vector<var\_decl>,

55  std::vector<statement> >(),

56  whitespace\_grammar<Iterator> >

57  derived\_var\_decls\_r;

58

59  boost::spirit::qi::rule<Iterator,

60  std::pair<std::vector<var\_decl>,

61  std::vector<statement> >(),

62  whitespace\_grammar<Iterator> >

63  generated\_var\_decls\_r;

64

65  boost::spirit::qi::rule<Iterator,

66  statement(),

67  whitespace\_grammar<Iterator> >

68  model\_r;

69

70  boost::spirit::qi::rule<Iterator,

71  std::vector<var\_decl>(),

72  whitespace\_grammar<Iterator> >

73  param\_var\_decls\_r;

74

75

76  boost::spirit::qi::rule<Iterator,

77  program(),

78  whitespace\_grammar<Iterator> >

79  program\_r;

80

81  };

82

83

84  }

85 }

86

87 #endif

stan::gm::program\_grammar::data\_var\_decls\_r

boost::spirit::qi::rule< Iterator, std::vector< var\_decl >), whitespace\_grammar< Iterator > > data\_var\_decls\_r

**Definition:** program\_grammar.hpp:45

stan::gm::statement

**Definition:** ast.hpp:634

stan::gm::program\_grammar::derived\_data\_var\_decls\_r

boost::spirit::qi::rule< Iterator, std::pair< std::vector< var\_decl >, std::vector< statement > >), whitespace\_grammar< Iterator > > derived\_data\_var\_decls\_r

**Definition:** program\_grammar.hpp:51

stan::gm::statement\_grammar

**Definition:** statement\_2\_grammar.hpp:19

functions\_grammar.hpp

expression\_grammar.hpp

stan::gm::expression\_grammar

**Definition:** expression07\_grammar.hpp:23

stan::gm::program\_grammar::model\_name\_

std::string model\_name\_

**Definition:** program\_grammar.hpp:30

statement\_grammar.hpp

stan::gm::program

**Definition:** ast.hpp:795

stan::gm::var\_decls\_grammar

**Definition:** var\_decls\_grammar.hpp:20

stan::gm::program\_grammar::derived\_var\_decls\_r

boost::spirit::qi::rule< Iterator, std::pair< std::vector< var\_decl >, std::vector< statement > >), whitespace\_grammar< Iterator > > derived\_var\_decls\_r

**Definition:** program\_grammar.hpp:57

stan::gm::program\_grammar::var\_map\_

variable\_map var\_map\_

**Definition:** program\_grammar.hpp:31

stan::gm::program\_grammar::program\_r

boost::spirit::qi::rule< Iterator, program(), whitespace\_grammar< Iterator > > program\_r

**Definition:** program\_grammar.hpp:79

stan::gm::program\_grammar::model\_r

boost::spirit::qi::rule< Iterator, statement(), whitespace\_grammar< Iterator > > model\_r

**Definition:** program\_grammar.hpp:68

stan::gm::variable\_map

**Definition:** ast.hpp:439

stan::gm::program\_grammar::program\_grammar

program\_grammar(const std::string &model\_name)

stan::gm::program\_grammar

**Definition:** program\_grammar.hpp:22

var\_decls\_grammar.hpp

stan::gm::program\_grammar::statement\_g

statement\_grammar< Iterator > statement\_g

**Definition:** program\_grammar.hpp:37

stan::gm::program\_grammar::error\_msgs\_

std::stringstream error\_msgs\_

**Definition:** program\_grammar.hpp:32

stan::gm::program\_grammar::functions\_g

functions\_grammar< Iterator > functions\_g

**Definition:** program\_grammar.hpp:38

ast.hpp

stan::gm::whitespace\_grammar

**Definition:** whitespace\_grammar.hpp:11

whitespace\_grammar.hpp

stan::gm::program\_grammar::generated\_var\_decls\_r

boost::spirit::qi::rule< Iterator, std::pair< std::vector< var\_decl >, std::vector< statement > >), whitespace\_grammar< Iterator > > generated\_var\_decls\_r

**Definition:** program\_grammar.hpp:63

stan::gm::program\_grammar::param\_var\_decls\_r

boost::spirit::qi::rule< Iterator, std::vector< var\_decl >), whitespace\_grammar< Iterator > > param\_var\_decls\_r

**Definition:** program\_grammar.hpp:73

stan::gm::program\_grammar::expression\_g

expression\_grammar< Iterator > expression\_g

**Definition:** program\_grammar.hpp:35

stan::gm::program\_grammar::var\_decls\_g

var\_decls\_grammar< Iterator > var\_decls\_g

**Definition:** program\_grammar.hpp:36

stan::gm::functions\_grammar

**Definition:** functions\_grammar.hpp:21


---

[ Stan Home Page ]

*© 2011–2014,
Stan Development Team.*
